# Predictive Evaluation of Microbiological Stability of Soft Drinks with *Lycium barbarum* L. Stored at Temperature Shifts

**DOI:** 10.3390/molecules27175508

**Published:** 2022-08-27

**Authors:** Aleksandra Plucińska, Aleksandra Marczak, Alina Kunicka-Styczyńska, Andrzej Baryga

**Affiliations:** 1 Department of Sugar Industry and Food Safety Management, Faculty of Biotechnology and Food Sciences, Lodz University of Technology, Wólczańska 171/173, 90-530 Łódź, Poland; 2 National Food Institute, Technical University of Denmark, 2800 Lyngby, Denmark

**Keywords:** goji berry, superfoods, food safety, polyphenols, prognostic microbiology

## Abstract

*Lycium barbarum* L., used in Chinese traditional medicine for centuries, has gained popularity in Europe in the last decade because of its health-promoting properties assigned to phenolic compounds and antioxidant activity. Goji fruits and extracts are often used as ingredients in popular homemade milk cocktails. Within this study, the microbiological stability of the milkshake, with the addition of berries from NingXia Province and their extract, was evaluated using the ComBase^®^ prognostic model. The extraction of dry berries in water at 70 °C for 72 h produced an extract showing radical inhibition of 64.9% and a total phenol content of 63.6 mg g^−1^. The phenolic compounds with the highest concentrations were in turn: 3-hydroxybenzoic acid, gallic acid, procyanidin B2, and catechin. The milkshake inoculated with the reference *B. subtilis* was a model for the study of its microbiological stability. Using ComBase^®^, a microbiological response to the delayed cooling of goji berry extract and the milkshake with the addition of goji berries was predicted and the model’s accuracy assessed. The best-performing models were constructed for extract (Bias factor *B_f_* 1.33, Accuracy factor *A_f_* 3.43) and milkshake (*B_f_* 1.29, *A_f_* 1.65) in a profile simulating delayed refrigeration (22.5 °C–9 °C–23 °C). Despite discrepancies between predicted and observed bacterial growth due to the antimicrobial effect of the derivatives of goji berries, the models were validated as „overpredict”, i.e., „fail safe”, and may be used to prognose the stability of these products in the given temperature profile.

## 1. Introduction

Goji berry *Lycium barbarum* L., commonly known as goji berry, may be an example of a superfood. Thanks to research carried out on the fruits of this shrub, many properties described for thousands of years regarding their pro-health characteristics have been confirmed [1,2]. The berries of *L. barbarum* are a rich reservoir of nutrients and are proven to be protective against retinal disorders, expressing a hypoglycemic effect. Moreover, they are characterized by antioxidant, immunostimulating, anticancer, and anti-atherosclerotic effects [3]. Goji berries are rich resources of polyphenolic compounds, with the most numerous flavonoids, and above all flavonols, including kaempferol, myricetin, and quercetin [4]. A diet rich in polyphenols is recommended for the prevention of many diseases, e.g., cardiovascular and neurodegenerative diseases, cancer, and diabetes. Polyphenols are also recognised as factors promoting the immune system, offering protection from UV radiation, and stimulating antimicrobial action [5,6,7,8,9]. The role of polyphenols in the removal of oxygen and reactive oxygen species (ROS) is also underlined [10] with their implications for cell proliferation and modulation of specific proteins [11]. Polyphenols’ anti-angiogenic and anti-inflammatory activity and deoxyribonucleic acid protection from ROS may result in ceasing the progression of tumors and triggering of apoptosis in tumor cells [12]. The antioxidant activity of flavonoids is mainly attributed to its ability to facilitating the chelating of metal ions [5]. The antibacterial effect of polyphenols is mainly associated with interactions with bacterial cell wall and cytoplasm membranes, leading to structural disorder of the cell and evoking cytoplasm leakage [13]. One way to enrich the diet with polyphenols is to use goji berries as food ingredients.

Most probably, *L. barbarum* is native to an area from south-eastern Europe to southwest Asia and was introduced to Europe in the 18th century [14]. Fruits of the species are small, about 2 cm long, red-orange ellipsoids with a sweet-tangy taste [15]. Commonly ripe fruits after harvesting are squeezed for juice, wine, or beer, cooked in medicinal Chinese herbal soups, teas, or dried [14]. In Europe, goji berries are usually distributed in a dried form and used as a raw material for food production. Drying procedures do not guarantee complete microbiological safety and fruits are vulnerable to microbial contamination, especially with xerophilic molds and spore-forming bacteria [16].

Predictive microbiology is a tool for assessing the microbiological risk of food products in order to prevent food spoilage and food-borne illnesses. Mathematical models describing the growth of microorganisms in food matrices combine knowledge about food-borne microorganisms, mathematics, statistics, information systems, and technology [17]. ComBase^®^ is an online platform for predictive microbiology, modeling the growth of bacterial populations under a particular combination of chosen environmental factors such as temperature, pH, and water activity [18]. The effect of the other stress factors e.g., packaging atmosphere, synthetic preservatives, and biologically active ingredients should be evaluated and the accuracy of the models validated by external validation, taking into account the actual response of the microorganism in the food product. The application of dried goji berries and their derivatives in soft drinks may substantially change the behavior of contaminating microorganisms with implications for safety assessments. Dried goji berries are valuable ingredients in low-processed health-promoting food products, and as the source of polyphenols and antioxidants, may serve as a factor in the microbiological stability of soft drinks. Homemade fruit extracts and milkshakes are often not refrigerated, or the cold chain is interrupted during their storage.

The aim of the study was to evaluate the microbiological stability of homemade goji berry extracts and milkshakes with dried goji berries subjected to discontinued refrigeration, applying a predictive microbiology tool. To model the spoilage of the bacteria *B. subtilis*, growth storage experiments were conducted in different temperature profiles, and constructed growth curves were compared to predictions of an online platform for predictive microbiology, ComBase^®^, and validated. Validated models can serve as an aid in assessing microbiological quality and safety in food products of similar characteristics to tested counterparts.

## 2. Results and Discussion

### 2.1. Phenolic Content and Antioxidant Activity in Goji Berry Water Extract

In traditional Chinese medicine, goji berries are commonly consumed in the form of mild Yin tonics, soups, and tea [19]. In Europe, goji berries have gained popularity as an ingredient of functional foods and are ungrudgingly consumed with muesli, milkshakes, and tea or water infusions. Homemade water–herbal extracts are usually prepared with hot water at the temperature of 70–98 °C [20]. Polyphenols express different temperature sensitivity but the degradation rates of some compounds may not vary substantially at 60 °C and 80 °C [21]. Thus, in the research we used an aqueous extract of dried berries after extraction at 70 °C for 72 h. Moreover, the extraction parameters were previously optimized to maximize the health-beneficial properties of the extract according to the total phenolic contents and the antioxidant activity (data not published).

The extract under the study was characterized by a total phenolic content (TPC) of 63.63 mg g^−1^ (Table 1).

The composition of plant extracts depends on many factors, including the type of solvent. Since literature data on aqueous goji berry extracts study are scarce, hydroalcoholic extracts were used for comparison. Hydromethanolic extracts of dried *L. barbarum* berries grown in Europe were characterized by a total phenolic content equal to 71.4 mg Trolox g^−1^ [22], while scientists studying fresh fruit determined the TEAC level at 85.8 mg Trolox per 100 g [23]. Literature data on the phenolic content of goji berries extracts differ significantly not only due to differences in research methodology, but above all due to the origin of the plant material, harvest time, and drying method. According to the literature, the content of phenols in dried fruit extracts falls in the range of 1.32–106.8 mg GAE g^−1^ dry extract [24,25,26,27,28,29,30,31]. Studies on the influence of the type of solvent used for extraction indicate a significant dispersion of the polyphenol level, from 14.13 to 109.72 mg GAE g^−1^ dry extract. The concentration of polyphenols was even eight times higher in the ethyl acetate extract compared to one with water used as the solvent [1]. The team of Skenderidis et al. [32] assessed the phenol content in goji berries grown in Europe and Asia. According to their findings, berries harvested in Greece were characterized by an average of 17% lower phenolic content than samples originated from China, where the TPC was estimated as 299.62 mg GAE g^−1^ extract from dried fruit.

Phenolic compounds identified in goji–water extract are presented in Table 1. Within the group of flavan-3-oles, catechin and procyanidin B2 were predominant and found in amounts of 376.474 and 372.751 µg g^−1^, respectively. The tested water–goji extract was also rich in quercetin-3-O-rutinoside, known as rutin (102.191 µg g^−^^1^), belonging to flavanols. Considering the identified phenolic acids, the highest content of 3-hydroxybenzoic acid (1425.604 µg g^−1^), *p*-coumaric acid (128.666 µg g^−1^), and gallic acid (792.925 µg g^−1^) was noted.

The catechin content of the tested goji berries extract was about 3.5 times lower than that recorded for dried goji berry by Protti et al. [33] and 27 times lower compared to the results of Pires et al. [22] for dried berries. The level of rutin in our extract, although the highest among the identified flavonols, was 11–162 times lower than that recorded in dried berries [22,33]. The quercetin-3-*O*-rhamnoside content in the water extract was only twice lower than reported for dried goji berry by Protti et al. [33]; however, the only traces of this compound were found in dried berries in other research [22]. The level of gallic acid in the extract was five times higher compared to fresh goji berries originating from Italy [34]. The high content of 3-hydroxybenzoic acid, also found in other fruits e.g., pineapples, bilberry, citrus can be attributed to its good solubility in water [35]. The content of phenolic compounds in water extract is determined not only by the level of individual compounds in dried goji berries, but above all by their solubility. In search of phenolic compounds in goji berries, mixtures of organic solvents and water are usually used, e.g., methanol/water acidified with HCl [34] or methanol/water [22,33]. Although the use of only water as a solvent significantly reduces the amount of phenolic compounds, it should be remembered that in the practice of home nutrition, aqueous extracts of dried goji berries are routinely prepared for direct consumption.

The goji berries–water extract shown the degree of radical inhibition at the level of 64.93% (Table 1). Strong antioxidant activity in goji berry extracts is usually associated with a high level of total phenolic content. Literature data on the antioxidant activity of goji berries vary due to different methods of determination and the way in which the results are presented. The antioxidant activity of Greek dried goji berries extract in 40% ethanol was presented as a Trolox equivalent and equal to 1831.9 mg TE L^−1^ [36]. Another study on three brands of dried goji berries expressed as Vitamin C equivalents showed their antioxidant activity from 54 to 61 µmol VCE g^−1^ [33]. Two samples of Chinese dried red goji berries originating from the NingXia region were characterized by antioxidant activity levels of 64.38 and 55.87 mg TE g^−1^ in the ABTS^•+^ radical scavenging assay [37]. Methanolic dried goji berries extracts, examined to scavenge DPPH radicals, expressed 81.38-83.7% of antioxidant activity [38].

### 2.2. Prediction of Soft Drinks with Goji Berries Stability

**Figure 1:**
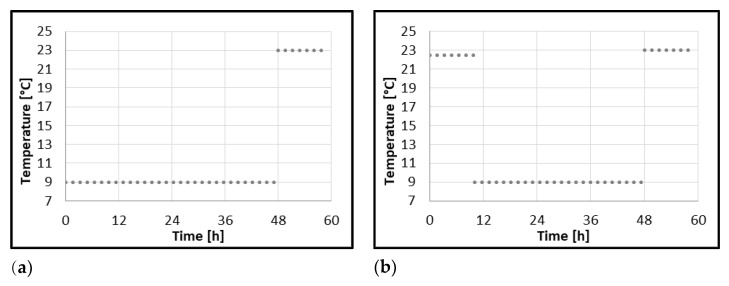
Temperature profiles of incubations of soft drinks containing goji berries: (**a**) Profile I 9 °C–23 °C for samples refrigerated immediately after inoculation, (**b**) Profile II 22.5 °C–9 °C–23 °C for samples refrigerated 10 h after inoculation.

*B. subtilis* is a common plant pathogen also associated with roots that promote plant growth via nitrogen fixation. The role of *B. subtilis* in the postharvest processing and flavor development of spontaneously fermented food is also known. Its resistance to desiccation and temperature treatments enable this bacteria to develop in low processed food products sourced from plant material. However, this species is regarded as a food-spoiling bacterium rather than a pathogen in reported incidences of food-associated illnesses caused by *B. subtilis* [39]. According to our previous research, the dried goji berries used in this experiment were not contaminated by strict pathogens. Since the only contaminants isolated were aerobic spore-forming bacteria *B. subtilis* (data not presented), this species was chosen for the presented model study. Models were developed of *B. subtilis* growth in dried goji berries–water extract and milkshakes with delayed refrigeration during their storage, applying the predictive microbiology platform ComBase^®^ [18]. Two different incubation profiles were constructed to simulate scenarios of temperature abuse in homemade preparations of goji berries. Profile I starts with incubation of samples at 9 °C for 48 h, followed by a 10 h incubation period at 23 °C, simulating the preparation of goji berry products, which are immediately refrigerated, taken out of the fridge after 48 h, and consumed after the following 10 h. Profile II begins with incubation at 22.5 °C for 10 h, followed by incubation at 9 °C for 38 h, and ending with a 10 h incubation period at 23 °C. This profile simulates goji berry products refrigerated after 10 h from preparation, stored in the fridge for 38 h, and consumed after 10 h from that point. Both profiles are shown in Figure 1.

It was found through a pairwise *t*-test that profile I, where goji berry soft drinks were refrigerated immediately, does not significantly promote the growth of *B. subtilis* (Table A1, Table A2, Table A3 and Table A4). On the other hand, there is a significant difference (*p* ≤ 0.012) between the observed maximum growth rate (μ_max_) for goji berry extract in profile I (Table A4) and the observed μ_max_ for extract in profile II (Table A5). The profile of delayed refrigeration (profile II) showed a trend of faster bacterial growth than the profile of samples of goji berry soft drinks that were immediately refrigerated (profile I). The trend can be explained by the lag phase that will be prolonged if incubation starts with refrigeration as in profile I. Even though a temperature rise occurred at the end of the refrigeration period, the growth of *B. subtilis* was slow and stable throughout the incubation. On the other hand, with the refrigeration delayed by 10 h, the lag phase was short or rapid growth occurred immediately after inoculation, and was further slowed down by refrigeration. In that profile, *B. subtilis* grew with a maximum growth rate µ_max_ 0.021 h^−1^ for the milkshake and µ_max_ 0.019 h^−1^ for the extract until its growth was slowed down by refrigeration.

**Figure 2:**
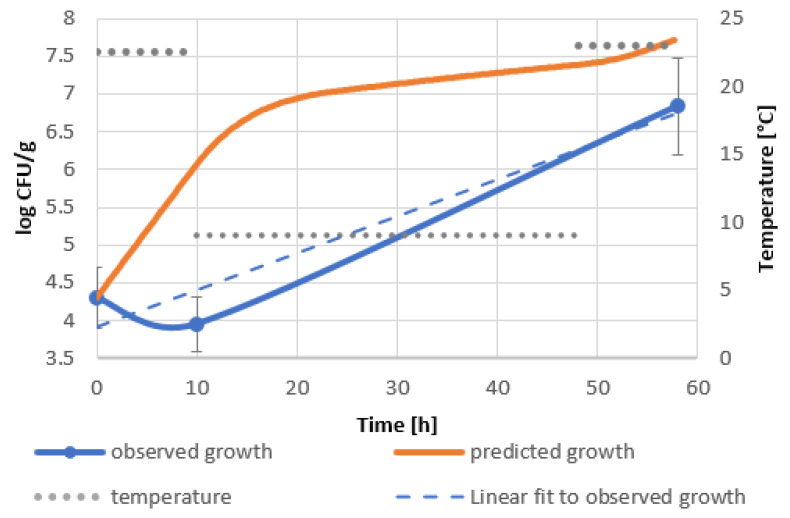
Predicted and real growth of *B. subtilis* in goji berry extract in temperature shifts, profile II 22.5 °C–9 °C–23 °C.

**Figure 3:**
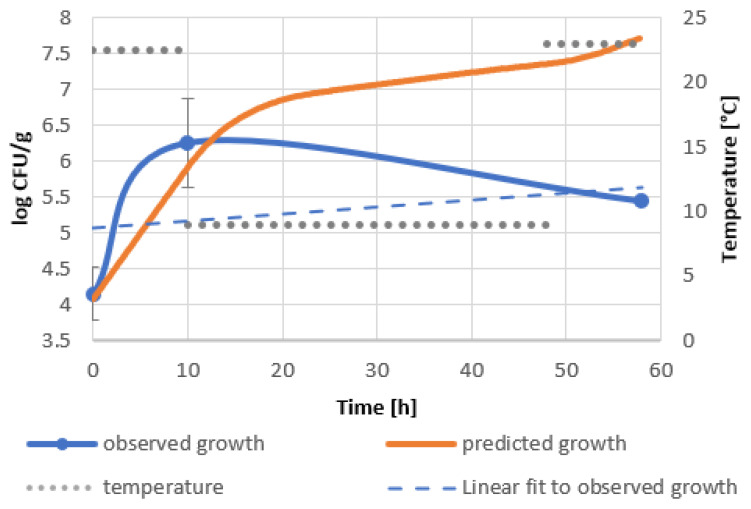
Predicted and real growth of *B. subtilis* in goji berry milkshakes in temperature shifts, profile II 22.5 °C–9 °C–23 °C.

Significant discrepancies between predicted and observed bacterial growth should be noted. The growth curve of the bacteria introduced into the goji berry extract shows the inhibition of bacterial growth within 10 h of incubation at 22.5 °C, unlike the model, which predicts an increase in bacterial level of about 1.5 logarithmic units (Figure 2). This phenomenon can be attributed to the antimicrobial effect of biologically active compounds of goji berry extract. The increase in the rate of bacterial growth during the cooling phase can be associated with the psychrophylic nature of the bacterial strain and its ability to degrade phenolic substances, including polyphenols [40,41]. Faster multiplication of *B. subtilis* in goji berry milkshakes in the first phase of profile II compared to the predicted one indicates the use of readily available carbon and nitrogen sources from milk and the low impact of biologically active substances introduced with goji berries. During refrigeration and its interruption by incubation at 23 °C, a decrease in the number of bacteria by almost 1 logarithmic unit was observed. As a result, the discrepancy between the actual bacterial level and the predicted one reached 2 logarithmic units (Figure 3). The decrease in bacterial viability may be due to the extraction of biologically active compounds from the mass of goji berries, rich not only in polyphenols, but also containing other biologically active substances [19,22] or their synergistic effect with milk compounds.

The best performing models were constructed for goji berry extract and milkshakes in profile II, which simulates delayed refrigeration, as shown in Figure 2 and Figure 3. Validation factors for all models are included in Table A6. The model’s precision was assessed by Bias (*B_f_*) and Accuracy (*A_f_*) factors regarded as quantitative measures of the model’s performance [42]. The Bias factor is a measure of overprediction (*B_f_* > 1) or underprediction (*B_f_* < 1) of the model [43]. Values in the range of 0.87–1.43 describe acceptable models. A range between 0.95 and 1.11 designates good predictions. The accuracy factor is a measure of precision and indicates how approximate predictions are in comparison to observed growth [44]. The validation of the model of goji berry extract storage (Figure 2, Table A6) showed *B_f_* equal to 1.33 and an *A_f_* of 3.43. The corresponding factors in the model of the goji berry milkshake storage (Figure 3, Table A6) were as follows: *B_f_* 1.29 and *A_f_* 1.65. Both of the models present acceptable Bias factors, which result in the models’ validation.

Predictive models in temperature-shift profiles can serve as an aid in managing the risk of spoilage or growth of pathogens throughout the food supply chain. Antolinos et al. [45] studied the growth kinetics of *Bacillus weihenstephanensis* in carrot soup and diluted cream pasta with temperature shifts 12 °C, 84 h–10 °C, 84 h–15 °C, 72 h. A bacterial growth pattern similar to the effect studied here of delayed refrigeration was observed for the predicting model. *B. weihenstephanensis* grew steadily up to 7 log CFU g^−1^ over the first 84 h, and in the next 84 h in lower temperature the growth was slowed down, followed by a final increase up to 15 °C in the last 72 h. On the other hand, the growth of *B. weihenstephanensis* was rapidly promoted by the rise in temperature. This trend is contrary to the pattern observed in our study, where an increase in temperature after an incubation period at 10 °C for 48 h had no significant effect on promoting the growth of *B. subtilis* both in goji berries extract and milkshakes with goji berries. This may be due to differences in physical states between model microorganisms, growth kinetics, or available nutrients in tested food preparations. Valero et al. [46] similarly studied the effect of temperature (8 °C, 12 °C, and 16 °C) changes and acidification on the growth of *B. cereus* in vegetable preparations. The specific bacterial growth rate increase was recorded as the temperature of incubation was increasing, opposite to our findings. Slowing down the growth of *B. subtilis* in the presence of *L. barbarum* derivatives may be attributed to the antimicrobial effect of polyphenols, expressing the ability to form complexes with external proteins, resulting in disturbances in the structure of cell walls [13] and cytoplasmic membranes [23,47].

Dried goji berries and their water extract were proven to be valuable compounds of soft drinks both due to the polyphenol contents, antioxidative activity, and antibacterial effects. Goji berry derivatives may contribute to preserving the microbiological stability of soft drinks subjected to temperature shifts during storage. In the food industry, standard shelf-life tests do not provide for the microbiological stability monitoring of products under interrupted cold chains. The proposed models can be used by food manufacturers to assess the products’ microbiological safety in the development of low-processed or unpreserved soft drinks with goji berry derivatives. Despite the positive validation of the presented models for goji berry milkshakes and the extract, they are limited to these specific matrices and temperature profile. The predicted growth of contaminating microorganisms in the prognostic microbiology models is usually higher than is experimentally observed [17], which is in agreement with our research. Extending the models to other storage conditions and extracts requires experimental testing. Moreover, given the still vivid trend of preparing homemade functional food, our research can be useful as information and educational materials aimed at individual consumers.

## 3. Materials and Methods

### 3.1. Dried Goji Berry Sample

Sun-dried goji berry fruits (*Lycium barbarum* L.) were harvested in NingXia province, China and obtained thanks to courtesy of NingXia Senmiao Technology and Development Co., Ltd. (Chengdu, China).

### 3.2. Extract of Goji Berries

The dry fruits of goji were powdered using a mortar. Three grams of berries were extracted with 30 mL of distilled water. The extraction time was 72 h, while the extraction temperature was 70 °C. The extract was centrifuged at 12,000 rpm for 15 min and the supernatant was collected and kept in a freezer at −50 °C (UF V 700, Binder GmbH, Tuttlingen, Germany) until further analysis.

### 3.3. Total Phenolic Content

The phenolic content of the dried fruit extract was determined using a modified standard method based on the Folin–Clocalteau phenol reagent [48] and spectrophotometric determination at 765 nm (V-1200, VWR International Sp. z o.o., Gdańsk, Poland). For this purpose, 250 µL of water and 160 µL of Folin–Clocalteau reagent were added to 300 µL of the extract and incubated for 5 min at room temperature. Then, 500 µL of 7.5% sodium carbonate solution was added to each of the samples and incubation was continued for a further 30 min. The sample was diluted 40-fold before taking the absorbance measurement. A control test was performed, replacing the standard solution with distilled water. The standard calibration curve was plotted using Trolox at concentrations of 0.01–0.1 mg·mL^−1^. The results were expressed in mg Trolox equivalents TEg^−1^ of dried fruit extract. The average of three replications with standard deviation was presented.

### 3.4. Phenolic Compounds Content

Chromatograph: HPLC + Ultimata 3000 Dionex (Thermo Fisher Scientific, Waltham, MA, USA). Column: Accucore C18 (150 mm × 3 mm; 2,6 µm) (Thermo Fisher Scientific, Waltham, MA, USA). Column temperature: 30 °C. Eluent A: HPLC purity water with the addition of 0.1% formic acid. Eluent B: acetonitrile with the addition of 0.1% formic acid. Flow rate: 0.35 mL/min. Detector: UV-Vis. Sample injection: 2 µL. Detection of hydroxybenzoic acids and flavan-3-ols was carried out at wavelength 280 nm. Hydroxycinnamic acid detection was carried out at wavelength 320 nm. The detection of flavonols was carried out at wavelength 365 nm. The identification of the phenolic compounds was performed based on standard compounds. All samples were tested in triplicate. The results were presented as the average of three replications with standard deviation.

### 3.5. Antioxidant Activity

The antioxidant activity of the extract was assessed using a modified method [33]. A solution of 40 mL of 5 µM ABTS^•+^ Radical was prepared, which was activated by adding 352 µL of 1.4 mM potassium persulfate. The mixture was kept in the dark at room temperature for 16 h to ensure that the reaction occurs completely. An ABTS radical solution was 20-fold diluted in acetate buffer. An amount of 3 mL of a diluted radical solution was placed into a spectrophotometric cuvette and 150 µL of an extract sample was added. The absorbance was measured at 732 nm after 6 min reaction, with extraction solvent used as blank. The data were presented as the percentage inhibition according to the Equation (1).

%A = (1 − A_sample_/A_blank_) × 100 (1)

The results were presented as the average of three replications with standard deviation.

### 3.6. Predictive Modelling

#### 3.6.1. Soft Drink Preparation

The water extrac tof dried goji berries was prepared as described in Section 3.2. For the preparation of the milkshake, 5 g of goji berries in proportion to 45 mL of milk were blended with a sterile blender to a uniform consistency. The pH of the beverages was measured with a pH meter (SevenExcellence pH meter S400, Mettler-Toledo Sp. z o.o., Warsaw, Poland), water activity was estimated for both goji berries extract and Tryptic Soy Broth medium (Merck, Darmstadt, Germany), and the salt content of the milkshake was calculated from the labeling information of the milk package. Parameters of the samples are shown in Table 2.

#### 3.6.2. Strain Activation

*Bacillus subtilis* strain ATCC 6633 originated from American Type Culture Collection was activated by passaging from TSA to TSB medium and incubation for 24 h in 30 °C, and subsequently for another 24 h in 30 °C. Activated strain culture 8 log CFU mL^−1^ was obtained.

#### 3.6.3. Soft Drink Inoculation by *B. subtilis* and Incubation

Samples of goji berry milkshake were inoculated by *B. subtilis* bacteria to achieve two different initial levels of contamination: 4.3 log CFU g^−1^ and 5.3 CFU g^−1^. Another set of samples with milkshakes was not inoculated for control. For goji berry extract samples, a 4.50% solution of extract in a TSB culture medium was prepared and inoculated analogously as milkshake samples. The initial level of bacterial culture was log 4.3 CFU g^−1^ and log 6.7 CFU g^−1^. For a control, a TSB medium was inoculated to achieve a comparable initial level of bacterial culture.

Different incubation profiles were constructed to simulate scenarios of temperature abuse in homemade preparations of goji berries; profile I: 9 °C for 48 h −23 °C for 10 h; profile II: 22.5 °C for 10 h, 9 °C for 38 h, 23 °C for 10 h. To compare the effect of refrigeration time on bacterial growth, another set of samples was incubated at 9 °C for 72 h. For recording viable count data from growth experiments, the count plate method with Tryptic Soy Agar—TSA (Merck, Darmstadt, Germany) was applied. The plates were incubated at 30 °C for 72 h. The number of microorganisms was expressed as the average of three replications with the standard deviation as CFU g^−1^. The growth was recorded at 3 points of time: for refrigerated samples: at 0 h directly after inoculation, 24 h, and 72 h. For samples in temperature profile I, the growth was recorded at 0 h, 24 h and 58 h. For samples in temperature profile II, the growth was recorded at 0h, 10 h and 58 h. The standard deviation of the mean concentration was calculated from duplicate milkshake samples. The average standard deviation was calculated to be 9.3% of the mean concentration, and it was assumed to be the same for the goji berry extract. Growth experiment data can be viewed in Table A1 and Table A2. Pairwise *t*-tests were conducted to investigate the difference between observed µ_max_ for samples in different incubation profiles.

#### 3.6.4. Construction of Predictive Growth Curves

Predictive growth curves were constructed with a web-based platform for predictive microbiology ComBase^®^ [18], in which the Broth Growth Model was chosen for modeling the response of *B. subtilis*. The physical state was assumed to be 8.5 × 10^−5^ CFU g^−1^ for refrigerated samples and in profile I, as it was expected that the lag phase could be prolonged by refrigeration, which decreases the physical state. The physical state of samples in profile II was assumed to be 1. Data recorded in growth experiments were fitted to a linear model in ComBase^®^ DM Fit to obtain the observed µ_max_. The initial level of *B. subtilis* was specified to the Broth Growth model of ComBase^®^ to obtain the predicted µ_max_. All predictions were then validated with the Bias factor and Accuracy factor. The accuracy and precision of the models were validated with the Bias factor (*B_f_*) and Accuracy factor (*A_f_*). The *A_f_* factor measures model precision. Formulas to calculate this are shown in Equations (2) and (3), according to Østergaard [44].

(2) $$B_{f}=10^{\left(\Sigma log\left(\frac{\mathrm{predicted}\mu_{\max}}{\mathrm{observed}\mu_{\max}}\right)/n\right)}$$

(3) $$A_{f}=10^{\left(\Sigma \left|log\left(\frac{\mathrm{predicted}\mu_{\max}}{\mathrm{observed}\mu_{\max}}\right)\right|/n\right)}$$

### 3.7. Statistical Analysis

The results of the total viable count of microorganisms, total phenolic contents, individual phenolic compounds, and antioxidant activity were expressed as the mean of three independent experiments with standard deviation. Statistical differences in prognostic modeling were evaluated by *t*-test with *p* ≤ 0.012.

## 4. Conclusions

The presented studies indicate a pronounced antibacterial effect of dried goji berries and their water extract in soft drinks stored under temperature shifts. The water extract of goji berries with total polyphenols value 63.63 mg g^−1^ and antioxidant activity of 64.93% (ABTS^•+^ radical inhibition) was characterized by the highest concentration of 3-hydroxybenzoic acid, gallic acid, procyanidin B2, and catechin. Although microbiological responses to temperature shifts of the drinks contaminated with *B. subtilis* experimentally assessed and simultaneously prognosed with ComBase^®^ online predictive microbiology tool varied, the models were validated as „overpredict”, i.e., „fail safe”, and may be used to prognose the stability of these products in the given temperature profile. The application of the models to other temperature profiles or other extracts requires experimental verification.

## Figures and Tables

**Table 1:** Phenolic compounds, total phenolic compounds, and antiradical efficacy of the goji berries–water extract; results are presented as an average value of three repetitions with ± SD.

Group	Compound	Contents [µg g^−1^]
Flavan-3-oles	Epigallocatechin	167.784 ± 0.004
	Catechin	376.474 ± 0.003
	Epicatechin	13.942 ± 0.001
	Epigallocatechin gallate	62.723 ± 0.012
	Procyanidin B2	372.751 ± 0.006
	Procyanidin C1	145.145 ± 0.003
Flavonols	Quercetin-3-*O*-glucoside	7.301 ± 0.003
	Quercetin-3-*O*-rutinoside (rutin)	102.191 ± 0.011
	Quercetin-3-*O*-rhamnoside	5.318 ± 0.008
	Quercetin	0.508 ± 0.001
	Myricetin	1.971 ± 0.002
	Kempferol	2.981 ± 0.001
	Luteolin-3-*O*-glucoside	0.288 ± 0.001
Hydroxycinnamic acids	Caffeic acid	52.508 ± 0.005
	Chlorogenic acid	16.259 ± 0.003
	*p*-Coumaric acid	128.666 ± 0.012
	Ferulic acid	13.890 ± 0.008
Hydroxybenzoic acids	Gallic acid	792.925 ± 0.002
	4-Hydroxybenzoic acid	55.612 ± 0.001
	3-Hydroxybenzoic acid	1425.604 ± 0.011
	Vanillic acid	98.055 ± 0.003
	Siringic acid	60.594 ± 0.002
	Protocatechic acid	55.363 ± 0.002
Total phenolic contents	[mg TE g^−1^]	63.63 ± 1.45
Antiradical efficacy	[%]	64.93 ± 0.47

**Table 2:** Chemical parameters of goji berries milkshake, goji berries extract, and TSB medium.

Soft Drink	pH	Water Activity	NaCl [%]
Goji berry milkshake	6.0	-	0.1
Goji berry extract	6.5	0.997	-
TSB medium	6.5	0.997	-

“-” not considered.

## Data Availability

Not applicable.

## Sample Availability

Samples of the compounds are available from the authors.

## Appendix A

**Table A1:** The average count of *B. subtilis* from growth experiments in different temperature profiles in goji berry extract and TSB growth medium.

Temperature Profile	Time [h]	CFU g^−1^	1.00 × 10^3^	1.00 × 10^5^	1.00 × 10^6^
9 °C	0	TSB	5	1	0
		TSB	>300	49	5
		extract	20	1	0
		extract	>300	61	0
	24	TSB	2	1	0
		TSB	1600	42	2
		extract	24	0	0
		extract	1200	32	6
	72	TSB	26	2	0
		TSB	NA	NA	164
		extract	22	0	0
		extract	NA	NA	192
I	0	TSB	5	1	0
		TSB	>300	49	5
		extract	20	1	0
		extract	>300	61	0
	24	TSB	2	1	0
		TSB	1600	42	2
		extract	24	0	0
		extract	1200	32	6
	58	TSB	NA	728	33
		TSB	NA	33	3
		extract	8	0	0
		extract	100	12	2
II	0	TSB	5	1	0
		TSB	>300	49	5
		extract	20	1	0
		extract	>300	61	0
	10	TSB	13	1	0
		TSB	>300	69	13
		extract	9	0	0
		extract	>300	113	14
	58	TSB	32	0	0
		TSB	100	3	0
		extract	NA	69	14
		extract	NA	128	28

NA—not applicable.

**Table A2:** The average count of *B. subtilis* from growth experiments in different temperature profiles in goji berry milkshake.

Temperature Profile	Incubation Time [h]	CFU g^−1^	1.00 × 10^2^	SD	1.00 × 10^3^	SD	1.00 × 10^5^	SD	1.00 × 10^6^	SD
9 °C	0	Control	>300	NA	5.0	0.0	2.0	0.0	2.0	0.0
		Milkshake	>300	NA	14.3	2.3	1.7	0.6	1.3	1.2
		Milkshake	>300	NA	223.3	54.9	2.0	1.7	2.0	0.0
	24	Control	>300	NA	8.0	1.4	2.0	1.4	0.0	0.0
		Milkshake	>300	NA	12.5	0.7	0.0	0.0	0.5	0.7
		Milkshake	>300	NA	155.0	0.0	4.0	2.8	0.5	0.7
	72	Control	59.0	0.0	13.0	0.0	0.0	0.0	1.0	0.0
		Milkshake	38.0	0.0	13.5	0.7	0.0	0.0	0.0	0.0
		Milkshake	>300	NA	196.5	33.2	1.0	0.0	0.5	0.7
I	0	Control	>300	NA	5.0	0.0	2.0	0.0	2.0	0.0
		Milkshake	>300	NA	14.3	2.3	1.7	0.6	1.3	1.2
		Milkshake	>300	NA	223.3	54.9	2.0	1.7	2.0	0.5
	24	Control	>300	NA	8.0	1.4	2.0	1.4	0.0	0.0
		Milkshake	>300	NA	12.5	0.7	0.0	0.0	0.5	0.7
		Milkshake	>300	NA	155.0	0.0	4.0	2.8	0.5	0.7
	58	Control	74.0	1.4	11.5	2.1	1.5	2.1	0.0	0.0
		Milkshake	143.0	0.0	8.0	1.4	1.0	1.4	0.0	0.0
		Milkshake	>300	NA	209.0	0.0	5.5	0.7	1.0	0.0
II	0	Control	>300	NA	5.0	0.0	2.0	0.0	2.0	0.0
		Milkshake	>300	NA	14.3	2.3	1.67	0.6	1.3	1.2
		Milkshake	>300	NA	223.3	54.9	2.0	1.7	2.0	0.0
	10	Control	>300	NA	>300	NA	6.0	5.7	1.0	0.0
		Milkshake	>300	NA	19.0	7.1	18.0	4.2	2.0	1.4
		Milkshake	>300	NA	17.0	0.0	1.0	0.0	0.0	0.0
	58	Control	>300	NA	86.0	7.1	3.0	2.8	0.0	0.0
		Milkshake	522.0	0.0	280.0	0.0	1.5	0.7	1.0	1.4
		Milkshake	>300	NA	>300	NA	25.0	0.0	2.5	0.7

SD—standard deviation, NA—not applicable.

**Table A3:** Observed and predicted values of maximum growth rate for refrigerated (9 °C) samples of goji berry milkshake, extract, and TSB medium.

Preparation	R^2^	Observed μ_max_ [h^−1^]	SD	Predicted μ_max_ [h^−1^]
Milkshake	−0.786	−0.000268	0.000773	0.021
Milkshake	−0.929	−0.000417	0.002170	0.021
Control	NA	0.046800	NA	NA
TSB	0.175	0.011800	0.009850	0.019
TSB	0.735	0.022800	0.008920	0.019
Extract	0.786	0.000357	0.001030	0.019
Extract	0.572	0.023000	0.012000	0.019

R^2^—coefficient of determination for linear fit to observed growth data, μ_max_—maximum growth rate, SD—standard deviation, NA—not applicable.

**Table A4:** Observed and predicted values of maximum growth rate for samples of goji berry milkshake, extract, and TSB medium in temperature profile I.

Preparation	R^2^	Observed μ_max_ [h^−1^]	SD	Predicted μ_max_ [h^−1^]	
				10 °C	23 °C
Milkshake	−0.980	0.000118	0.001180	0.021	0.198
Milkshake	−0.988	−0.000228	0.002910	0.021	0.198
Control	0.996	0.001890	0.000089	NA	NA
TSB	1.000	−0.002930	0.000007	0.019	0.191
TSB	0.474	0.069700	0.039000	0.019	0.191
Extract	0.804	−0.007380	0.004840	0.019	0.191
Extract	0.849	−0.031800	0.009080	0.019	0.191

R^2^—coefficient of determination for linear fit to observed growth data, μ_max_—maximum growth rate, SD—standard deviation, NA—not applicable.

**Table A5:** Observed and predicted values of maximum growth rate for samples of goji berry milkshake, extract, and TSB medium in temperature profile II.

Preparation	R^2^	Observed μ_max_ [h^−1^]	SD	Predicted μ_max_ [h^−1^]		
				22.5 °C	10 °C	23 °C
Milkshake	0.988	0.009870	0.032700	0.184	0.021	0.198
Milkshake	0.162	0.026700	0.022700	0.184	0.021	0.198
Control	0.861	0.008900	0.032500	NA	NA	NA
TSB	0.740	0.012200	0.004710	0.178	0.019	0.191
TSB	0.891	−0.032000	0.007690	0.178	0.019	0.191
Extract	0.854	0.049000	0.013700	0.178	0.019	0.191
Extract	0.154	0.004170	0.003570	0.178	0.019	0.191

R^2^—coefficient of determination for linear fit to observed growth data, μ_max_—maximum growth rate, SD—standard deviation, NA—not applicable.

**Table A6:** Bias factor (*B_f_*) and Accuracy factor (*A_f_*) for TSB medium, goji berry milkshake, and goji berry extract in temperature profile I and in temperature profile II.

Temperature Profile	TSB Medium		Milkshake		Extract	
	*B_f_*	*A_f_*	*B_f_*	*A_f_*	*B_f_*	*A_f_*
9 °C	1.16	1.39	NA	NA	6.63	8.03
I	0.37	2.72	177.97	177.97	NA	NA
II	1.56	1.56	1.29	1.65	1.33	3.43

NA—not applicable.
